# Unseen, unheard: a qualitative analysis of women’s experiences of exclusively expressing breast milk

**DOI:** 10.1186/s12884-022-04388-6

**Published:** 2022-01-21

**Authors:** Lisa A. Anders, Karen Robinson, Jennifer M. Ohlendorf, Lisa Hanson

**Affiliations:** 1 University of North Carolina at Greensboro School of Nursing, Nursing and Instructional Building, 1007 Walker Avenue, Greensboro, NC 27412 USA; 2 Marquette University College of Nursing, 510 N 16th St, Milwaukee, WI 53233 USA

**Keywords:** Human milk, Breast feeding, Breast milk expression, Lactation, Bottle feeding, Qualitative research

## Abstract

**Background:**

Breast milk feeding has numerous benefits for women and infants. Positive maternal experiences with breast milk feeding impacts exclusivity, duration, and maternal mental health. Most research focuses on women feeding directly at the breast. Some women elect to feed exclusively expressed milk to their healthy, term infants rather than feed directly at the breast. Little is known about what constitutes a positive experience among this population. Therefore**,** the aim of this study was to explore women’s experiences of exclusive expression (EE).

**Methods:**

Interviews were conducted via Microsoft Teams to collect qualitative data from a purposive sample of 21 women practicing EE. Interviews were analyzed for themes.

**Results:**

Three themes: *Unseen and Unheard, Doing it My Way, and Getting into the Groove*, and 8 subthemes: *Breast is Best, Missed Opportunities for Healthcare Provider Support, Fighting for it, What Works for Us, A Sense of Control, Preparation, Tricks of the Trade, and Making it Manageable* were identified. Despite challenges, including a lack of support from healthcare providers and a lack of acknowledgement as breastfeeding mothers, exclusive expression offered participants a method to continue breast milk feeding in a way that they found to be satisfying.

**Conclusion:**

This study provides insight into experiences of exclusive expression that clinicians can use to improve their support of breast milk feeding during perinatal encounters. Societal pressure to feed from the breast may have negative emotional consequences for women electing to exclusively express. There is a need for more information and support for breast milk expression from healthcare providers along with a reframing of how breast milk feeding is discussed and promoted.

## Background

It has been well-established that infants who receive breast milk and women who produce milk have improved health outcomes compared to infant formula [1–3]. Because of the many of the benefits of breast milk, major health organizations recommend exclusive breast milk feeding (feeding only breast milk with no formula, water, solid or semi-solid foods) for the first 6 months of life [3–5]. In the US, only a quarter of infants exclusively receive breast milk for 6 months, falling well short of the World Health Organization (WHO) targets of 50% by 2025 [6]. Nationally, exclusivity rates descend from 47% of infants receiving exclusive breast milk feeding (BMF) at 3-months to 26% at 6-months [7].

Positive maternal experiences and emotions have been shown to be associated with higher rates of exclusive BMF and longer duration. For instance, higher scores on the Maternal Breastfeeding Evaluation Scale (MBFES), a tool that includes aspects of satisfaction and enjoyment, was associated with greater likelihood of exclusive breastfeeding at 5 months [8]. In this scale’s initial development, high levels of satisfaction were found to be predictive of breastfeeding duration [9]. Additionally, positive emotions during breastfeeding and positive maternal attitudes toward breastfeeding have both been associated with exclusive BMF outcomes [10–12]. If healthcare providers can assist women in achieving a satisfying feeding experience, they may be able to influence BMF duration and exclusivity.

Unfortunately, what is known about breastmilk feeding satisfaction has been focused direct breastfeeding (DBF), defined as feeding an infant latched at breast. For instance, the MBFES was designed based on experiences of successful BMF from the breast and contains language specific to DBF [9]. Therefore, it is difficult to generalize the findings to those practicing other methods of breast milk feeding as this can also occur through expression of breast milk that is bottle-fed to the infant. The practice of breast milk expression is common, and globally, there is an increasing number of women who practice exclusive expression (EE), in which infants are exclusively fed breast milk that is expressed without latching to the breast [13, 14]. There are few studies examining the experience of EE. In a study of women who breastfed directly and expressed milk occasionally, the experiences of breastmilk feeding were different in that the body work and labor of breastfeeding directly gave a sense of satisfaction while expressing milk was challenging, stressful, and in some instances “not worth the time” (p. 74) [15]. In a previous study conducted by Anders et al. (unpublished observations), the experiences of participants who exclusively expressed were compared to those who fed directly at the breast. All participants found satisfaction in providing breast milk to their infants. Participants who ultimately practiced EE despite intentions to DBF were left with subsequent feelings of frustration and guilt that their experience did not match their intent. While EE allowed them to provide breast milk while negotiating early postpartum fatigue and latch problems, they experienced a lack of support from healthcare providers and negative feelings towards their bodies that they viewed as failing. Because many participants practicing EE in this study had prenatal intentions to DBF, these findings are not transferrable to persons with intentions to practice EE. There is a scarcity of literature related to satisfaction for persons intending to practice EE from birth.

If healthcare providers are to support a sustained and satisfying BMF experience for women who practice EE, there is a need to understand the experience from the maternal perspective. Moreover, it has been suggested that research focusing on how women carry out and think about the act of BMF, in other words, an embodied perspective, may offer insight into BMF duration [16]. Therefore, the aims of this study were to explore the experiences of persons who practice EE with the specific research questions: a) What is the feeding experience for women who EE? b) How do women describe their infant feeding goals and intentions? and c) What factors influence women’s experiences of infant feeding?

### Feminist theory

This research was conducted from a feminist philosophical perspective. Some researchers who use feminist theory have noted that breastfeeding can be viewed as empowering, a way of embracing the nature of the female body [17, 18]. However, breastfeeding promotion research has focused on what is best for the infant [19], ignoring the experiences for those providing the milk. Stearns notes that this can be seen as a “gift to the infant rather than as the product of mother’s embodied labor”(p. 17) [15]. This medicalization and moralization of breastfeeding promotion centered on the needs of the infant has brought critique from feminist scholars and liberal feminists; Placing the infants’ needs at the center of breastfeeding reduces women’s sovereignty over their own bodies, may ignore the importance of their own personal fulfillment, and does not acknowledge the social constraints impacting feeding choices [17, 19]. Additionally, societal norms of breastfeeding as feeding directly at breast have placed limitations on how women define their infant feeding experiences as shown by Anders et al. (unpublished observations).

The unique voices of women who choose to practice EE is not widely represented in current societal, medical, or research definitions of breastfeeding. As such, care must be taken when constructing knowledge of EE to not further reduce the value of the individuality of women’s experiences by imposing societal breastfeeding goals and interpretations. Therefore, research from a feminist philosophical perspective is necessary to ensure that the voices of women whose infant feeding method has been traditionally underrepresented in the breastfeeding literature are heard.

## Methods

### Ethical considerations

Ethical approval for this study was obtained from the Marquette University Institutional Review Board. Participants were provided and read an information sheet and verbal consent was recorded prior to beginning the interviews.

### Study design

This study employed a qualitative design with narrative analysis. Narrative methods are well-suited for research from a feminist epistemological standpoint because detailed stories of participants’ experiences can be used to gain insight into phenomena that have been suppressed in scientific research [20, 21], as is the case for those exclusively feeding breast milk via exclusive expression to term infants. After participants shared their stories, probing questions (Table 1) were used as needed to obtain deeper understanding [21].

**Table 1:** Probing Question Examples

“Tell me about…”	
“… how you made decisions about feeding your baby”	
“… your feelings about pumping and feeding pumped^a^ milk”	
“…your daily life while pumping^a^ for your baby?”	
“…your relationships with family and friends during your pumping^a^ experience	

^a^ Participants and the general population from which the sample came from used the terms pump rather than expression

### Participants and setting

The target population for this study was women who had given birth and practiced exclusive expression within the past year. To obtain varied perspectives from a diverse sample, participants were purposively sampled from Facebook groups for persons who express breast milk. The first author (LA) selected open and closed Facebook groups, requested access and permission to post study information from group administrators, and joined the groups for the purpose of recruitment. Between September-November 2020, a flyer containing a link to an eligibility screening survey was posted to the selected groups whose administrators had granted permission. Those expressing interest were eligible to participate if they were age 18 or older, could speak and read English, had given birth to a term (greater than or equal to 37 weeks gestation), singleton infant within the past year, and had exclusively fed their own breast milk for at least 2 weeks using exclusive expression. Two groups, those who intended to EE and those who intended to directly breastfeed but ultimately arrived at EE, were invited to participate in this study. Exclusion criteria included parents of multiples and parents or infants with complications including prematurity or requiring Neonatal Intensive Care Unit admission.

### Data collection

LA conducted interviews via Microsoft Teams between September and November of 2020. Microsoft Teams is a videoconference software that also allows for audio only calls. Participants chose whether they wanted to use video or audio only to respect their wishes for privacy. Participants were read a consent form and gave verbal consent to be interviewed and audio recorded. Demographic data was collected using closed questions that were read to participants while LA recorded verbal responses. Participants were then asked to share their story of infant feeding experiences. After the storytelling phase of the interview, the conversational phase included asking probing questions as needed to obtain more information and gather a holistic impression of the experience of breastmilk feeding. Probing questions were developed from the literature and concepts from the Revised Situation-Specific Theory of Breastfeeding, a theory that recognizes the holistic experience of BMF and emphasizes the “personal and embodied reality of the experience” [22]. Examples of probing questions are listed in Table 1.

### Data analysis

The transcribed interviews were read in their entirety by LA and KR to gain familiarity with the text and to confirm accuracy of the data. An inductive thematic analysis of the narratives [23, 24] began with development of an initial coding schema by each investigator independently after reading the transcripts. Code generation and refinement continued throughout the analysis with data management using NVivo software. Codes were then organized and classified into themes. The two investigators discussed codes and themes to reach a consensus. Comparisons were made between groups and within groups [25] to identify commonalities and differences in infant feeding experiences and behaviors based on feeding intention. Member checking with five of the participants provided confirmation of the investigators’ interpretations. These participants were chosen at random, called by LA, provided with the synthesized and analyzed data in the form of themes, and were encouraged to offer discussion, new data, or dispute the interpretations [26]. Demographic data were analyzed using descriptive statistics.

## Results

### Sample characteristics

Demographic characteristics of the sample (*N* = 21) are presented in Table 2.

**Table 2:** Participant Demographics

	Intention to EE	Did Not Intend to EE	Total
	*n* = 10 (%)	*n* = 11 (%)	*n* = 21 (%)
Race			
Asian	2 (20)	1 (9)	3 (14)
Black/African American	2 (20)	6 (55)	8 (38)
Hispanic	0 (0)	1 (9)	1 (5)
White, Non-Hispanic	5 (50)	3 (27)	8 (38)
Mixed- Race	1^a^ (10)	0 (0)	1 (5)
Marital Status			
Single	3 (30)	3 (27)	6 (29)
Married	7 (70)	8 (73)	15 (71)
Level of Education			
High school diploma	1 (10)	1 (9)	2 (10)
Some College	2 (20)	2 (18)	4 (19)
College Degree	4 (40)	4 (36)	8 (38)
Graduate school or higher	3 (30)	4 (36)	7 (33)
Income			
Under $20,000	0 (0)	1 (9)	1 (5)
$20,001-40,000	1 (10)	2 (18)	3 (14)
$40,001-60,000	4 (40)	0 (0)	4 (19)
$60,001-80,000	1 (10)	2 (18)	3 (14)
$80,000 and above	4 (40)	6 (55)	10 (48)
Parity			
Primiparous	6 (60)	9 (82)	15 (71)
Multiparous	4 (40)	2 (18)	6 (29)
Birth			
Vaginal	8 (80)	9 (82)	17 (81)
Planned Cesarean	1 (10)	1 (9)	2 (10)
Unplanned Cesarean	1 (10)	1 (9)	2 (10)

^a^ Participant self-identified as Black and White

About half (*n* = 10) had intended to practice EE while the rest (*n* = 11) intended to either direct breast feed (DBF) or use a combination of feeding methods. Of the multiparous participants (*n* = 6), four had practiced EE previously and had intentions to repeat with the current newborn, two had practiced EE previously or a combination and had not intended to EE breast milk this time. The average duration of exclusive breast milk feeding was 5.04 months (SD = 1.20) with 5 participants still exclusively feeding breast milk at the time of the interview. All participants were still feeding at least some breast milk at the time of the interview. The infant age at the time of the interviews ranged from 2.75 months to 10.75 months (M = 6.41, SD = 2.51).

### Themes

**Fig. 1:**
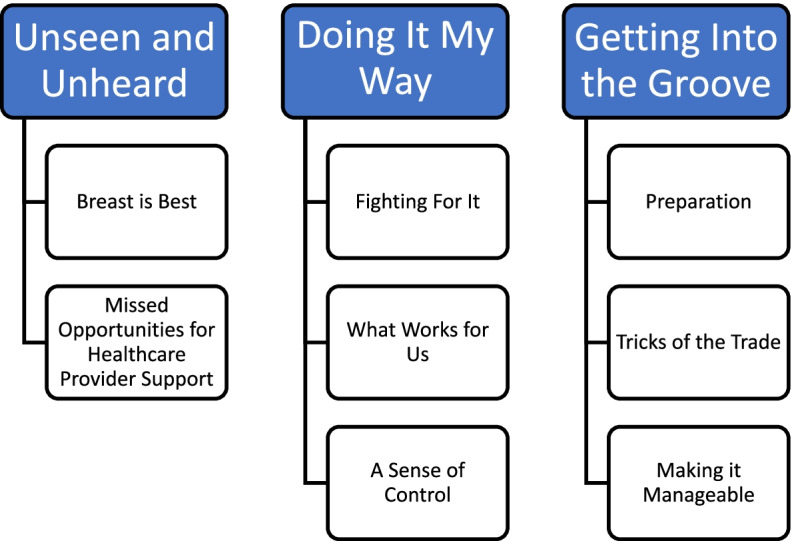
Themes and Subthemes

Three themes *Unseen and Unheard, Doing it My Way, and Getting into the Groove*, along with 8 subthemes: *Breast is Best, Missed Opportunities for Healthcare Provider Support, Fighting for it, What Works for Us, A Sense of Control, Preparation, Tricks of the Trade, and Making it Manageable* were identified and are shown in Fig. 1.

The first theme reflects participants’ experiences of the events and encounters that made participants feel that their concerns and support needs were not listened to and that they were not seen as breastfeeding mothers. The remaining two detail how they were able to continue practicing EE and how this method of feeding met their needs.

#### Unseen and unheard

##### Breast is best

Participants felt pressure from a variety of sources to not only feed breast milk, but to feed from the breast directly. Their goal of providing breast milk was not heard by others and often times the participants didn’t feel as though they were seen as “breastfeeding mothers.” This messaging started prenatally for participants that intended to EE. Even though it was one participant’s “plan to start pumping. I went to a breastfeeding class and felt that it was like my only choice to try to breastfeed after that” while she eventually stuck to her plan, the lack of acknowledgement of her plan as an option led to “guilt of not being able to directly breastfeed”. Several participants echoed the feeling that EE was not presented as an option. For others this continued in the hospital as

The lactation consultants at the hospital where it was like breastfeeding or nothing. Not nothing, of course you feed the baby, but there was this like sense of you can just keep nursing. Just keep nursing, just keep nursing. and it was like it didn’t matter.

Another who planned to EE agreed that her “opinion [to exclusively express] didn’t matter in the beginning” when hospital staff advised to her latch the infant directly to the breast.

When it was not their intention to EE, this pressure to get baby to the breast was “really hard, I feel like society kind of today expects us to breastfeed, and when we can’t we feel like failures” when direct breastfeeding was not working. One participant described a friend’s suicidal thoughts, which she believed stemmed from her unsuccessful breastfeeding experience due to the statement “breast is best”. She felt unheard when she was “telling her [obstetrician] it [direct breastfeeding] is not working”. She described her experience as “frustrating” when her provider “continued to push breastfeeding”.

Several participants described provider interactions that left participants feeling like they were not seen fully as breastfeeding mothers. When asked by the pediatrician “do you breastfeed or do you formula feed? I have to, you know, there ain’t no options for pump and I give him breast milk through the bottle, and they always look at you so crazy”. Another felt like the healthcare providers did not “know her [infant] as well just because that little checkbox [exclusive expression] isn’t on there.” One described these situations as making her feel like she was being “downgraded” while another compared the feeling to baseball players “who are taking steroids, they have an asterisk on them.” Feeding their infants in public brought scrutiny when they were seen feeding with a bottle. “People are like, ‘Oh, you should be breastfeeding.’ They don’t realize that I am.”

This judgement sometimes came with information that these participants found to be false such as “not giving them all the antibodies which of course isn’t true, and like you’re not gonna have like the bonding with the baby, and I just haven’t found really any of that to be true.” Even family and friends told participants that they were not really breastfeeding and asked repeatedly if they would try latched even when they intended to and carried out EE from the start.

##### Missed opportunities for healthcare provider support

Healthcare providers’ focus on the “breast is best’ led to what participants perceived as a lack of support for their chosen methods or implications that it was outside of the norm. Subsequently, healthcare providers missed valuable opportunities to provide education on breast milk expression. One participant noted that if she hadn’t “researched it on my own, I probably would be exclusively formula feeding. I think there’s a big opportunity for moms to note there is a viable third option.” Others reported that they were not provided education or demonstration of pump usage because they were told not to express during breastfeeding classes. When education regarding pumping was provided, it left participants feeling discouraged. For example, an obstetrician told one participant that the “milk wouldn’t be tailored to the baby since the saliva isn’t getting on the nipple.” Some participants, however, spoke of experiences that made them feel supported, being “cheered on” and reassured by nurses when carrying out her intentions of EE from the start. Others received valuable information about pump operation or how to ensure that pump parts were fitted properly to the breast in cases when different sizes were necessary.

Both participants who intended to EE and those that did not felt that at times, providers did not support participants’ overarching goal of exclusively providing breast milk. When participants did not want to or had difficulty feeding directly at the breast, providers instead offered formula as the alternative. For instance, when one participant had difficulty latching,

They were kind of just like baby needs to eat. Like if you're not gonna feed her we need to give her a bottle [formula], and I was like you're not giving her a bottle [of formula]. I didn't ask for a bottle. I asked for help to see if I'm doing this right, and before we left the hospital, I ended up or they let me, they brought me a pump and I ended up pumping and feeding her from the bottle.

Another participant who did not feel supported in her decision to express when she was sent home with formula “just in case” EE didn’t work out.

Similarly, hospital staff offered formula when supplementation was needed instead of breast pumps or donor milk. One participant described her baby as “too sleepy to latch” while in the hospital. When she asked for donor breast milk, she reports being met with eye rolling from staff. The participant instead hand expressed until later requesting a breast pump from a different staff member. Some infants were jaundiced after birth and needed increased intake. For one participant who started with direct breastfeeding, a lactation consultant “scared the bejesus out of me [participant]” when she urgently told her “He is sleepy. He is jaundiced! He is not getting all the nutrients he needs. You need to give him formula right now” without mention of the option of giving expressed milk. She felt as though using formula was the only option until she went home and started expressing.

### Doing it my way

#### Fighting for it

Participants described EE as a challenging way to provide breast milk. From the start, those who had planned to EE fought through tensions with others over their decision. In response to the push to DBF or use formula, one stated that “Luckily I was strong enough in my own personal will” while another described having to “really push back on them”. A participant who struggled against her husband’s desire to give infant formula waited for him to leave the house so she could throw away the formula. While it was worth it to be able to feed breast milk rather than formula, they had to “put the effort into it. It’s kind of like exercise, if you put the time and dedication into it, you know not everyone has time for it. You have to want it, you know, and I obviously wanted it.” By putting in this effort in the beginning, they were able to continue.

After the initial fight to start, participants also found it difficult to continue EE. They described the time-consuming nature of pumping and cleaning pump parts. They also noted additional expenses related to pumping such as accessories to make the process hands-free, storage items such as breast milk storage bags and additional freezers, and replacement or extra pump parts. As milk needed to be expressed several times each day to avoid complications like clogged ducts, mastitis, or decreased supply, participants felt reliant on the pump.

Bringing in an adequate milk supply also meant “kicking it into high gear” by expressing every two to 3 hours which felt like a lot of work This time commitment is similar to DBF but with additional time for set-up and cleaning of pump supplies. Despite the time needed, participants described gratification in their accomplishments. “It just gave me a sense of pride that I didn’t give up . . . you know, in spite of the challenges, I was still able to do this for her and it felt like a necessary sacrifice.” The accomplishment of being able to provide breast milk made them feel that fighting through the challenges was worth it.

#### What works for us

Participants shared a variety of pragmatic experiences that led to or reinforced their EE experience. For those that did not intend to practice EE, expressing worked better than latching directly at the breast. While some had pain or nipple damage from latch attempts, others found they did not enjoy feeding directly at the breast as they had expected. Participants made statements such as “it [DBF] just wasn’t this amazing experience”, “I didn’t enjoy it”, and thought “maybe this isn’t for me”. One participant who used EE with her last child felt more comfortable and choosing EE again. She described herself as lacking confidence when attempting to latch as she had only ever expressed previously. Another “fell into pumping” as she just started expressing more and more and found she did not miss direct breastfeeding. For others who intended to EE, it was a way that worked to avoid the difficulties they anticipated with direct breastfeeding such as latch problems due to flat nipples. They used the word “stressful” to describe thoughts of trying to latch and decided to bypass it by using EE. Even though EE was not the mainstream method for providing breast milk, it’s a way that worked for these participants’ lifestyles and bodies.

Participants described that EE gave them enhanced freedom to share feeding responsibilities. In their lives, it worked to have others help with feedings making it feel like a “team sport.” EE provided them with an opportunity for others to feed and bond with the infant. “It’s important to my family to keep us as bonded as we want to be. It allows my [older] daughter for the most part to get interaction with her [the infant].” While initially some worried that EE would not work for them because they thought they would miss out on bonding themselves, found that the route didn’t matter and bonding still worked for them as one participant stated:

Whether you're nursing or whether you're pumping, there will be moments where you feel like a cafeteria, you know, and then your sense of self is consumed by your sense of motherhood. It doesn't matter how you're doing it, how you're feeding your baby, you’re still going to feel like you don't know who you are anymore. Like that there's this loss of individual identity because now my identity is inevitably, inextricably, permanently tied with hers . . . but you're trading it for this sort of profound connection that you have with your baby.

In addition to the positive feelings that many expressed about facilitating these bonds, some also knew they “would be going back to work” and would “have to pump there anyway.” Exclusively expressing eased the transition as one participant did not “have to get her [infant] off the breast” after a 3 week maternity leave and another wanted to “rotate through who was doing the middle of the night feedings” to minimize fatigue upon returning to work.

#### A sense of control

Providing breast milk in this way gave them a sense of control and ownership over the process and in some cases their bodies. Participants spoke of tracking their output of milk along with their infant’s intake which gave them reassurance in knowing that their infants were receiving enough. “I have that reassurance of doing the math at the end of the day and knowing how much he’s getting.” One participant expressed that she had anxiety surrounding her infants’ intake and another also said it benefitted her psychologically to have knowledge of how much her infant was eating. This control carried over into their schedules as well with some describing structured routines that gave them control over when milk was expressed rather than feeding when the infant is hungry as in direct breastfeeding. Keeping track of and controlling their supply of milk allowed participants to plan when they could stop expressing but continue to provide stored milk.

EE also gave some participants a sense of bodily control and autonomy. When meeting resistance with her chosen infant feeding method, one stated “it’s my body and I’ll choose what I want to do with it” while another was uncomfortable with the thought of a direct latch and others touching her breasts to assist with latching stating “at some point this is my body too.” Another echoed the discomfort with the repurposing of her breasts after already experiencing the transition of pregnancy: “my body is mine and you know, your breasts are, for me, were used like in sexual ways and then having a baby and it’s going from you know like a body change.” For others, the need for this control over their breasts was limited to feeding in public as EE provided freedom from having to “always have to be like here out in public and whip out my boob”.

#### Getting into the groove

##### Preparation

Participants described various ways that they became physically and mentally prepared for feeding breast milk. With an intention to EE, some began research and preparations during pregnancy. Yet, with extremely limited access to resources and information, one felt that “we were figuring it out by ourselves along the way”. Those who attended breastfeeding classes received little information about milk expression. Therefore, social media proved to be a valuable information source. One said that knowledge gained made her “feel more empowered” and another stated knowing that “exclusively pumping existed was already a bonus point for me ‘cause I’ve read stories of some women who didn’t know that exclusively pumping was a thing at all.” For those who had practiced EE with a previous child, their prior positive experience made them feel prepared and confident.

For others who did not know of EE as an option and had not envisioned needing to express, research occurred after the infant was born. For one participant this meant having to supplement with formula, until she found the necessary information to begin EE. She described that this was difficult because resources she trusted, such as pediatrician and CDC websites, lacked information on EE. Doing this preparation and research after the infant was born left participants to “figure it out by ourselves along the way.”

##### Learning the tricks of the trade

Along the way, participants described learning about equipment and tips through a combination of advice from social media groups and their own trial and error experiences. Choosing pumps was an important decision during the journey. Common factors in determining which pump suited each individual were portability and suction. Pumps with adequate suction were necessary to avoid complications like plugged ducts, mastitis, or low milk supply. One participant noted that some pumps are made for only occasional use, not the frequent use that EE requires and were therefore not appropriate. Newer, wearable pumps that allowed users to EE “hands-free” offered time-savings and the freedom to perform other tasks which was important for participants with other children to care for or worked in settings that made milk expression during work hours challenging. Participants watched YouTube videos, read Instagram reviews, and asked questions in Facebook groups to gather EE tips and learn about equipment. They also learned about recommended pumping frequency and techniques like power pumping to establish supply from online support communities. One participant stated that “every person’s journey is different. .. and just kind of listening to my body” was an important part of the learning process.

##### Making it manageable

Exclusive expression was viewed as time-consuming and hard work, especially in the early weeks when expressing 8 to 12 times per day to establish a good milk supply. At times it felt all-consuming but once it became part of the routine of daily life “it didn’t feel like it was such a daunting, demanding thing” anymore. This was initially made more challenging for those without previous experience:

At the beginning I had to get in the groove of it because it was something I had not done ever and you kind of have to build a routine with it because it was very, very stressful at the beginning to know that you’re going to commit so much time a day to pump every two to three hours.

Because the beginning of the EE journey was challenging, many used small, step-wise rather than long-term goals for breast milk feeding duration. Once reaching a goal such as three-months, they would assess the manageability to decide on the next goal. The initial burdensome feeling of “being tied to a machine” later turned into a time to “kind of decompress” when participants started to distract themselves doing activities on their phones or watching streaming videos. For all participants continuing to EE seemed more manageable after “dropping pumps,” meaning that once the milk supply was established, participants would decrease the number of milk expression sessions per day, oftentimes dropping the “middle of the night pump” to get more sleep at night. The majority of the participants described having an oversupply of milk that allowed for “dropping pumps” without having to sacrifice exclusive breast milk feeding. Participants with an oversupply often described storing milk that allowed them to wean from EE but continue to feed breast milk and in some cases donate excess breast milk. The prospect of discontinuing EE with a supply of milk stored to feed also made the task of expression seem workable.

Even if I choose to stop pumping literally today that I still have four months’ worth of milk in my freezer so that’s beyond a blessing to me… It just made me want to do it more because I knew I could supply everything that she needed.

The ability to donate some of the surplus of breast milk was a rewarding experience. For example:

It really gives you great joy to like, be able to give someone something that they are not able to either produce or like I said for adoption, and you know, and I gave it away without you know much, maybe some milk bags in return, but it is very, very rewarding.

The COVID-19 Pandemic influenced the manageability of BMF. While in the beginning the pandemic was seen as an obstacle to obtaining support for latching, it later assisted in maintaining a manageable breast milk expression routine. Some participants were able to work from home once they returned to paid employment, a situation that they thought made expressing easier to accomplish.

Working from home I can work on the couch. I could work at my desk and work from the bed, and I do have like a pumping set-up, but it just gives me a lot more flexibility of course. If I was in my office, even though it's just 20 minutes a day, that's 20 minutes with the door locked. But it's also I have to get, you know, get it cleaned in the kitchen is not on my floor and all of that is just there would be so much more it would be so much more difficult if I had to be in the office for two of those pumps a day.

Another participant worked previously with children in various schools and homes and commented that if not for working remotely that she “probably have to get an electric pump and pump in my car or find another way to pump or not even be able to pump at all”.

Additionally, participants felt that they were able to maintain their milk expression daily routines without much difference on weekends due to stay at home orders. As one said, “pretty much the only places that I do go to are my doctor, her doctor, or my parents’ house, so there hasn’t really been a time where I don’t need, or I don’t have access to my pump”. This enabled her to have a “concrete schedule with everything that’s going on.” Another also saw this as a benefit stating “before we were always on the go, and I don’t know how I would be able to pump doing that. So, I’m actually really happy that we’re kind of in this quarantine.”

## Discussion

This study provides a unique contribution to the literature on breast milk feeding by detailing the maternal experience of EE for term infants. An overarching theme was that participants felt unseen and unheard stemming from pressure to feed at the breast and subsequently received little support for their method from healthcare providers. Promoting only exclusive direct breastfeeding as the superior method of infant feeding and focusing on the health benefits ignores the realities that women face [27] and creates a dichotomy leaving women feeling guilty when that specific, narrow breastfeeding goal is not met [28–32]. Even those who did meet their breast milk feeding goals via EE voiced feelings of guilt and did attempt latching due to this pressure.

Dietrich Leurer et al. found that participants who had ever expressed at all reported feeling negatively judged by healthcare providers for feeding expressed milk [33]. Some participants in the current study echoed similar sentiments. It has been suggested that “creating an equal platform for all feeding options” (p. 203) [34] may reduce these types of negative feelings. The ways that society and healthcare providers define and talk about breastfeeding may impact the experience of infant feeding especially for those who choose feed outside of societal norms.

These findings of these studies together could encourage those caring for breast milk feeding families to examine on their own knowledge and perceptions about the practice of EE to address this issue. Reflection on current feeding discussion with families could spark change could improve these interactions.

Pressure to feed at breast is also an example of the moralization of infant feeding that has been critiqued by feminist scholars [17]. Participants in this study were able to strike a balance between being empowered by their ability to produce milk and the constraints of the role of feeding the infant. Although at times they felt “tied to the pump” similar to DBF women, participants who EE also felt freedom to leave or return to work knowing their baby could easily be fed their breast milk by others. This made them feel that the transition to work was a smooth one. McCarter-Spaulding notes that direct breastfeeding from a liberal feminist stance is a “gender difference that stands in the way of liberating women” (p. 207) [18]. Furthermore, in the U.S. there is no paid maternity leave requirement which places social constraints on women who want to provide breast milk. EE is a method that feminist scholars and healthcare providers need to acknowledge in the face of these social constraints. The shared responsibility of infant feeding experienced by women who EE could be seen from this stance as a more equitable form of providing breast milk and allow for greater autonomy to be separated from their infants.

Participants sought information and support for breast milk expression on online platforms such as Facebook, Instagram, and YouTube. This is not unexpected given that the sample was recruited through the use of such online support communities. However, in participants’ own research, they found little information on EE from pediatricians or reputable sources such as library resources or healthcare organization websites. This is similar to findings by Strauch and colleagues that information sources included online parenting forums and one-way information websites and blogs but did not find any scholarly articles examining support for EE [35]. Similarly, Dietrich Leurer and colleagues also found that for women needing to express milk at all, milk expression information needs were often not addressed by healthcare providers [33]. With respect to EE, there are few information sources or classes available leaving many to do their own research from websites and online forums [36] which creates a vacuum of quality, reliable information. Healthcare professionals could take this as an opportunity to extend their influence into spaces where families are seeking support. This would fill the vacuum and promote health.

When discussing their goals and intentions, the overarching goal of participants in this study was to feed breast milk to their infants. To them, the route of delivery was less important than what was being fed. They also described step-wise and lengthening goals as their journeys proceeded based on manageability at each step. This reinforces findings that women find the prospect of exclusive breast milk feeding for 6 months daunting and have desires to view each breast milk feed as important [27]. Healthcare providers can celebrate and encourage patients in each step of their breast milk feeding journeys while discussing strategies to manage the demands of breast milk feeding.

All participants were still feeding breast milk to their infants ranging from 2.5 months of age to almost 11 months of age, many with plans to continue to 1 year or beyond. Existing literature identifies exclusive expression as a risk factor for early weaning [13, 37]. Yet these studies did not measure intentions or levels of support for EE. Participants in this study were able to meet their breast milk feeding goals via EE. Future quantitative analyses could compare DBF to EE in regard to duration of exclusive breast milk feeding for a larger population. More studies are needed to identify social and contextual factors involved in early weaning in this population to examine whether the relationships between EE and breast milk feeding duration persist when adequate EE education, anticipatory guidance, and support are present. In the meantime, healthcare providers should individualize their infant feeding conversations with families to account for individual contexts and barriers that may affect feeding decisions and duration.

## Strengths and limitations

This study is unique in its focus on the experience of only those who practiced EE for term infants. Therefore, a strength of this study is the inclusion of voices that have not previously been included in the literature on infant feeding. All 21 participants practiced EE for term infants while most literature on exclusive expression is either limited to special population such as premature or NICU populations or include those who express at all rather than practicing EE. Additionally, 62% of this sample were non-white making for an ethnically diverse sample.

Despite these strengths, there were limitations that restrict the transferability of the findings. First, this was a socioeconomically homogenous sample as many of the participants in this study were highly educated, married women, from households with middle to high income. Participants spoke of the supplies that they had to buy that were necessary to EE, such as different pumps, undergarments, and storage supplies. This may have made for a different experience for those from a less advantaged situation that may be unable to afford such amenities. More studies are needed with samples including women from broader socioeconomic backgrounds are needed as their experiences may different. Second, this study took place during the COVID-19 pandemic. Many participants noted impacts of the pandemic on professional and social support systems and employment situations that had effects on their EE journeys. There is evidence that during the pandemic, some mothers felt that breastfeeding was protected while others felt that breastfeeding was more difficult due to challenges obtaining support, feelings of isolation, and concerns related to breastmilk safety [38]. Therefore, these are context-specific findings, and further research on experiences of EE outside of a pandemic.

## Conclusion and clinical implications

While more studies are needed on the experience and practice of EE, providers can use this information to provide better support to families that feed expressed milk. Providers should assess their knowledge of breast milk expression and how they provide breast milk feeding information to families in their care. Inclusive language acknowledging all methods of feeding breast milk should be utilized and care should be taken to recognize the goals and unique needs of each family. Feeding directly at the breast may not be feasible or desired by all parents, but EE may be a viable option for some to provide breast milk. Parents should be informed and supported in these feeding decisions by their healthcare providers throughout the perinatal experience in order to adequately prepare for and execute their feeding plans.

## Data Availability

The data analyzed during the current study are not publicly available to protect the privacy and confidentiality of participants but are available from the corresponding author on reasonable request.

## Abbreviations

BMF: Breast milk feeding
DBF: Direct breastfeeding
EE: Exclusive Expression
